# 

**Published:** 1824-09-01

**Authors:** 


5. Simulated Diseases .. .. .. .. .. .. .. .. .. .. 508

6. Medical Remuneration .. .. .. .. .. .. .. .. .. 509

7. Supposed Punctured Wounds of the Diaphragm .. .. 510

8. Defamation of Character .. .. .. .. .. .. .. .. 511

XIII.

Bibliographical Record .. .. .. .. .. .. .. .. .. .. 512

XIV.

EXTRA-LIMITES.

I. Mr. Searle's Instrument for applying Pressure .. .. .. 516

II. Dr. Gordon Smith's Queries .. .. .. .. .. .. .. 519

III. Mr. Battley on the Components of Opium.. .. .. .. 521

XV.

INTELLIGENCE, &c.

I. Society of Physicians of the United Kingdom .. .. .. 523

II. Artificial Seltzer Water .. .. .. .. .. .. .. .. 525

III. Literary Intelligence .. .. .. .. .. .. .. .. 525

IV. Protection of Vaccination.. .. .. .. .. .. .. .. 525

List of Additional Subscribers .. .. .. .. .. .. .. 526

---

### TO SUBSCRIBERS.

Much inconvenience having been experienced in consequence of this Journal not being published on the regular quarter days, it is determined, that, in future, it shall appear on the 1st. of January, 1st. April, 1st. July, and 1st. October. No. 3, therefore, will be published on the 1st. of January, 1825, instead of 1st. December, 1824.

### XIII.

### BIBLIOGRAPHICAL RECORD;

OR,

*Works received for Review since last Quarter.*

1. A comparative View of Fever and Inflammatory Complaints, with Essays illustrative of the Seat, Nature, and Origin of Fever. By THOMAS MILLS, M. D. Licentiate of the King and Queen's College of Physicians Octavo, pp. 125. Dublin and London, April, 1824.

---

2. Revue Medicale Française et Etrangère; et Journal de Clinique de l'Hotel Dieu et de la Charité de Paris. Avril, 1824. (In exchange.)

---

3. An Introduction to Anatomy and Physiology. For the Use of Medical Students and Men of Letters. By THOMAS SANDWITH, Surgeon, Octavo, pp. 192. London, 1824.

---

4. Observations on Fever. By R. WADE, Member of the Royal College of Surgeons, and Apothecary to the Westminster General Dispensary. 8vo. pp. 83. London, June, 1824.

☞ *This is addressed to "the notice of students," but will not be found undeserving the attention of some who have long quitted that rank.*

---

5. Strictures on "Mr. Pattison's Reply to certain Oral and Written Criticisms. By W. GIBSON, M.D. Philadelphia, 1820. Octavo, sewed, pp. 48.

---

6. The Institutes and Practice of Surgery:—being the Outlines of a Course of Lectures. By WILLIAM GIBSON, M.D. Professor of Surgery in the University of Pensylvania; Surgeon and Clinical Lecturer to the Alms-House Infirmary, &c. Vol. 1, 8vo. pp. 470. Plates. Philadelphia, 1824.

☞ *We hope to be able to notice this first volume from the able pen of Dr. Gibson very shortly.*

---

7. Physiological Views of the Structure, Functions, and Disorders of the Stomach and Alimentary Organs of the Human Body, with Observations on the Qualities and Effects of Food and Fermented Liquors, and on the Influence of Climate and Local Situation. By THOMAS HARE, F.L.S. F.H.S. Fellow of the Royal College of Surgeons in London, &c. 8vo. pp. 368, 2 plates. Second Edition. London, June, 1824.

---

8. An Elementary System of Physiology. By JOHN BOSTOCK, M.D. F.R.S. L.S. and H.S. &c. Vol. 1, pp. 518. London, May, 1824.

☞ *We are glad to see an original elementary work on Physiology from the pen of an Englishman. Dr. Bostock appears capable of doing justice to the subject. We should suppose there will be two—at all events, one, volume more. We shall shortly enter on a review of the publication, which we have no hesitation, in the mean time, to recommend strongly.*

---

9. Evils of Quarantine Laws, and Non-existence of Contagion; deduced from the Phenomena of the Plague of the Levant, the Yellow Fever of Spain, and the Cholera Morbus of Asia. By CHARLES MACLEAN, M.D. 8vo. pp. 444. London, June, 1824. [*In our next.*]

---

10. A Voyage to India: containing Reflections on a Voyage to Madras and Bengal, in 1821, in the Ship Lonach; Instructions for the Preservation of Health in Indian Climates; and Hints to Surgeons and Owners of Private Trading Ships. By JAMES WALLACE, Surgeon of the Lonach. 8vo. pp. 166. London, June, 1824.

☞ *The instructions and hints contain good and wholesome advice; and we can conscientiously recommend this little volume to those to whom it is more particularly addressed. Of the non-medical or descriptive portion of the volume, we could also speak favourably, were we permitted—but, alas! from such flowery paths of literature we are now severed—perhaps for ever!—We honour the head and heart of the author.* Vive valeque.

---

11. Official Report on the Fever which appeared on Board His Majesty's Ship Bann, on the Coast of Africa; and amongst the Detachment of Royal Marines, forming the Garrison of the Island of Ascension, in the year 1823. By WILLIAM BURNETT, M.D. one of the Commissioners of the Medical Department of His Majesty's Navy, Physician in Ordinary to his Royal Highness the Duke of Clarence, and Honorary Fellow of the Imperial Medico-Chirurgical Academy of St. Petersburgh. 8vo. pp. 78. London, June, 1824.

---

12. Elements of Physiology. By A. RICHERAND, Professor of the Faculty of Medicine of Paris, Member of the Academies of Vienna, Petersburgh, Madrid, Turin, &c. Translated from the French by E. M. DE LYS, M.D. Fourth Edition, with Notes and a copious Appendix. By JAMES COPLAND, M.D. Lecturer on Physiology, Pathology and Therapeutics; Consulting Physician to Queen Charlotte's Lying-in Hospital; Physician to the Royal Universal Infirmary for Children; and Member of the Royal College of Physicians, London, &c. &c. 8vo. pp. 704. London, July, 1824.

---

13. A View of the Formation, Discipline, and Economy of Armies; with an Appendix, containing Hints for Medical Arrangement in Actual War. By ROBERT JACKSON, M.D. A New Edition much enlarged. Quarto, pp. 545. Stockton and London, July, 1824.

---

14. Elements of Phrenology. By GEORGE COMBE, President of the Phrenological Society. With two Engravings. Duodecimo, pp. 227. Edinburgh and London, July, 1824.

---

15. A Summary of Physiology. By F. MAGENDIE, M.D. Translated from the French by JOHN REVERE, M.D. &c. Second Edition. 8vo. pp. 444 Baltimore, 1824.

16. Surgical Essays. By Baron D. J. Larrey, &c. Translated from the French, by John Revere, M.D. &c. 8vo. pp. 335. Baltimore, 1823.

☞ *Both the above works are very respectably translated from the original language, and we wish them success in transatlantic regions.*

---

17. The New York Medical and Physical Journal. Numbers 6, 7, 8, and 9. (In Exchange.)

☞ *This new Journal, dedicated almost exclusively, to American original communications, continues to increase in value as it proceeds.*

---

18. Observations on the Nature and Treatment of Fevers and Bowel Complaints which Travellers in Greece are exposed to: including Remarks on Climate, Malaria, the Safest Period of the Year for Travelling, and Hints for the Preservation of Health. Intended as a Medical Guide to Travellers. By John Somers Down, M.D. &c. &c. Resident Physician at Florence. Small 8vo. pp. 76. Rowland Hunter, St. Paul's Church-Yard.

☞ *This little work is written in the form of familiar letters, and is well deserving the attention of every traveller through Italy and Greece.*

---

19. Istoria D'Una Specie Straadinaria de Cecita Conjuncta. Signior Antonio Scarpa. 1824.

---

20. Sull' Ottalmia che Hanno Sofferto i Militari di Livorno Osservationi. Di Lodovico Paoli. 8vo. 1824.

---

21. Memoria Sopra un Nuovo Strumento per Operare le Catarette e per Formare la Pupilla Artificiale. M. Georgi. 8vo sewed, 1822.

---

22. Storia della Malattia per la quale Mori il Conte G. Perticari. Giacomo Tommasini. Duodecimo, sewed, 1823.

---

23. Appendix to the Formulary for the Preparation and Mode of Employing several New Remedies, &c. &c. By Robley Dunglison, M.D. F.R.S. Nancy, &c. Translated from the French. 8vo. sewed, pp. 48. August, 1824.

---

24. Illustrations of the Arteries connected with Aneurism, and Surgical Operations. The Plates are intended to explain the relative Position of the Arteries, in respect to the Surrounding Parts, and the Organs to be met with in such Operations, both externally and internally to their Sheaths. This Plate is the first of a complete Series. By G. D. Dermott, M.R. C.S. Price Five Shillings; or Mounted in Boards Six Shillings. Burgess and Hill, July, 1824.

☞ *We wish Mr. Dermott, who is a zealous and indefatigable private teacher, every success in his undertaking.*

---

25. Anatomical Drawings, from Preparations in the Museum of the Army Medical Department at Chatham. Royal Folio, Nine Plates, and numerous Figures, with explanatory Letter Press. August, 1824.

☞ *We understand the Army Medical Museum at Chatham, is already rich in Pathological Specimens, and is rapidly increasing. We shall give some account of the specimens in this fasciculus in our next Number.*

26. The Westminster Review, No. 3, for July, 1824.

☞ *There is in this Number, an excellent article on the difficulty of procuring dead bodies for dissection. It will do much good through a popular medium like the Westminster Review.*

---

27. Original Cases, with Dissections and Observations Illustrating the Use of the Stethoscope and Percussion in the Diagnosis of Diseases of the Chest; also, Commentaries on the same Subjects; selected and translated from Avenbrugger, Corvisart, Laennec and others. Pp. xxxii. 336. Three Plates. By JOHN FORBES, M.D. Physician to the Chichester Dispensary. London, 1824.

---

28. Beobachtung einer Chroniscen Entzundung des Ruckenmacks mit ungewöhnlichem Ausgange nebst Bemurkengen daruber von LUDWIG WOLFF, Jun. M. et Ch. Dr. ausubendem Arzte zu Hamburg. Hamburg, 1824, pp. 151.

☞ *In this volume, a brief but yet interesting and practical view is taken of Inflammation of the Spinal Marrow. Some ingenious observations are also made upon Fungus Hematodes. We shall take a future opportunity of noticing the opinions of the ingenious author more at length.*

---

29. A System of Anatomical Plates; accompanied with Descriptions and Physiological, Pathological, and Surgical Observations. By JOHN LIZARS, F.R.S. E. Fellow of the Royal College of Surgeons, and Lecturer on Anatomy and Physiology. Edinburgh. Part V. Muscles and Joints of the Upper and Lower Extremities.

☞ *"This Part is accompanied with two supplemental Plates to Part* IV. *illustrative of Hernia, from dissections by the hand of Sir Astley Cooper, Bart. Surgeon to the King, &c. Kindly presented to the author, and preserved in his Museum at Edinburgh." This Part contains nine Plates, and* 136 *pages of Letter Press, price* 10*s.* 6*d.* Vires acquiret eundo.

---

30. Medical and Surgical Cases, selected during a Practice of Thirty-eight Years. By EDWARD SUTLEFFE, Queen Street, London. 8vo. pp. 628. August, 1824.

"In every work regard the writer's end."

---

31. Elements of the Etiology and Philosophy of Epidemics. In two Parts. By JOSEPH MATHER SMITH, M.D. Fellow of the College of Physicians and Surgeons of the University of the State of New York, &c. 8vo. pp. 223. New York, 1824. *Received* 18*th August*, 1824.

☞ *We return many thanks to Dr. J. Watts, Jun. for this acceptable present, and the accompanying letter.*

---

### TO SUBSCRIBERS.

It was our intention to have published a spare title page with each volume for the holders of the former, or Analytical Series; but on enquiry, we are led to think, that few of them would make use of it, as the size of the volumes of this Series will not correspond with that of the last, and as the blending of the two Series might create confusion in future references. We advise our Subscribers therefore, to bind and letter this Series as it now stands, keeping it distinct from the former; those who are of a different opinion, have only to make application through the channel of their booksellers, and spare title pages will be immediately furnished.

### *Additional Subscribers since last Quarter.*

A.

Alexander, Dr. Surgeon to the Nat. L.I. Hon. East India Company's Service, and Principal Medical Officer at the Prince of Wales' Island.

Alexander, Mr. William—to Jamaica.

Anderson, Dr. A. D. Member of the Royal College of Surgeons of London, St. Vincent Place, Gl asgow.

B.

Bell, Dr. Wakefield, Yorkshire.

Bell, Mr. J. Surgeon, Cape Coast Castle.

Betton, Samuel, M. D. German Town, near Philadelphia.

Bohan, Mr. Surgeon, 63d. Regt.

Buckingham, Mr. George, Hon. East India Company's Marine Service.

C.

Chirnside, Dr. Somerset Street, Manchester Square.

Colvan, Mr. Surgeon, Armagh, Ireland.

Conway, Mr. Henry Jones.

D.

Deans, Mr. Surgeon, Cheshire.

Dingwall, Mr. George (Student)

Dods, Dr. Bath.

Donaldson's Library, Brighton.

E.

Edwards, Dr. Evan, Wrexham, Denbighshire

H.

Haffenden, Thomas, Esq. Surg. Ashford, Kent.

Hart, Henry, M. D.

I. & J.

Innes, Mr. Charles, Student, Edinburgh.

Jewel, Mr. G. Surgeon-Accoucheur to the Westminster Institution for Diseases of Women and Children, Gerrard Street, Soho.

K.

Kerrison, Mr. George.

L.

Lavies, Mr. J. Surgeon, Charles Street, Westminster.

Leslie, William, Esq. Surg. to the Aberdeen General Dispensary.

M.

Mann, Mr. Robert, Member of the Royal College of Surgeons, Manchester.

Marshall, Mr. Assistant Surgeon, Royal Navy.

P.

Probart, Mr. Edward.

R.

Roberts, Mr. Charles Edwards—To East Indies.

S.

Seaforth, Mr. (Student)

Selwyn, Mr. Congreve, Surgeon, Ledbury, Herefordshire.

T.

Taylor, Mr. Student in Medicine.

V.

Vance, George, Esq. 27, Sackville Street.

W.

Waters, Mr. Allan, Exmouth, Devon

Whitefield, William, Esq. Surg. Ashford, Kent.

CHANGE OF RESIDENCE.

Dr. James Johnson, removed from Spring Gardens, to *Suffolk Place, Pall Mall East.*

### INDEX.

Abdominal inflammation, opium in 118
Abercrombie, Dr. on diseases of the heart 72
Abortion, artificial, its causes 341
Absence of the spinal marrow 4
Acetate of quinine 52
Acetum colchici, in hydrocephalic fever 365
Action of medicines, Dr. Kinglake on 225
Acute peritonitis 200
Alcock, Mr. on fractures of the patella and olecranon 112
Alum, saturated solution of, as a styptic 142
Ammonia in drunkenness 229
Amputation of the lower jaw 213
Amputation for tetanus traumaticus 217
Anatomy of the ear, on the 42
Anderson, Dr. on tobacco in tetanus 90
Andral, Dr. on diseases of the bronchia 451
Anemia, curious case of, by Dr. Coombe 295
Angina pectoris, Dr. Ryan on 203
Anomalous diseases of the spine 313
Antimonial powder, inert 227
Antimony, in large doses, in pneumonia 221
Apoplexy, meningeal, Dr. Johnson on 204
Apothecaries' Act, curious trial at York 243
Apothecaries' Act, remarks on 505
Apothecaries' Transactions reviewed 112
Arachnitis spinalis 30
Arachnitis cured 458
Armstrong, Dr. on opium in inflammation 118
Army medical fund, celebration of 252
Arsenic, Dr. Paris on 319
Arsenic, various tests for 321
Ascites complicated with pregnancy 500, 492
Atrophy of the spinal marrow 7
Auriculo-ventricular contractions 193
Auscultation, advantages of 197
Ballingall, Dr. on syphilis 85
Bampfield, Mr. obtains the Fothergillian medal 251
Battley, Mr. on the components of opium 253, 521
Beatty, Dr. letter from, on premature labour 357
Beck, Dr. on infanticide 337
Belladonna, preventive of scarlatina 490
Bifid brain, Dr. Duncan's case of 297
Bladder, sloughing of, post partum 235
Blindness, temporary 438
Blood, Dr. Scudamore on 137
Bloodletting, on the best mode of 141
Bowen, Dr. on cataract 396
Boyle, Mr. on the treatment of syphilis 172
Brain, on the functions of the 179
Brain, Dr. Lallemand on the pathology of the 265
Brain, Dr. Kellie on the pathology of the 285
Bronchia, on organic changes in the 451
Brown's, Mr. case of paracentesis capitis 214
Buchanan, Mr. on the ear 42
Buffy coat of blood 142
Bushell, Mr. on sulphate of quinine 226
Bushell, Mr. on rupture of the fallopian tube 234
Bye-law of the College of Surgeons 241
Cæsarean section, self-performed 236
Cæsarean operation successful 494
Calculi, dissolved by galvanism 223
Callaway, Mr. case of priapism 214
Callow, Mr. on obstructed colon 125
Capitis paracentis, in hydrocephalus 214
Carbonate of iron in neuralgia 198
Carbonic acid gas in the blood 138
Cardiac disease, M. Breschet on 188
Cases of premature labour, cured by mercury 357-8
Causes of artificial abortion 341
Cephalalgia, severe case of, cured by quinine 226
Cheselden's mode of lithotomy 207
Chevalier, Mr. on effusion in the spinal canal 23
Children, suffocation of 351
Children, drowning, hanging, strangulation of 351
Chisholm, Dr. on melena 231
Chisholm, Dr. on tape-worm 232
Cholera morbus of India, Mr. Cormick on 481
Chronic peritonitis 202
Cicuta, poisoning by 505
Cinchona, salts of 51
Classification of poisons 317
Clerk, Dr. his answer to Tommasini 166
Coagulation of the blood, Dr. Scudamore on 137
Colchicum, fatal effects of 53
Colchicum, Dr. Kinglake on 224
Colchicum, in tape-worm 232
Cold, on the fatal effects of, on two people 265
College of Surgeons, bye-law of 241
Colon, cases of obstruction in the 125
Combe, Dr. on anemia 295
Compression of the spinal marrow 1[illegible]

Concussion of the spinal marrow 15
Concussion and compression, Sir A. Cooper on .............. 466
Constipation, fatal in pregnancy 233
Cormick, Mr. on cholera of India 481
Crampton, Dr. on tinea ........ 359
Crampton, Dr. on chorea ...... 363
Cubebs, in gonorrhœa and rheumatism .................... 228
Cunningham, Mr. on the treatment of syphilis ............ 173
Curvature of the spine, Dr. Jarrold on .................... 303
Cut throat, Mr. Haden on ...... 132
Davis, Dr. his essay on phlegmatia dolens .................. 378
Debility, Dr. Shearman on...... 391
Defamation of character........ 511
Delivery, sudden .............. 496
Diagnosis of aortic aneurisms .. 183
Diagnosis of valvular disease of the heart ................. 196
Diaphragm, supposed wounds of 510
Diarrhœa, inflammatory........ 112
Diathesis, Rassori's observations on ...................... 222
Dickson, Dr. on sulphate of quinine ...................... 331
Dissolution of calculi by galvanism ...................... 223
Dods, Dr. A. on contorted spine 92
Dropsy, Dr. Stoker on ........ 144
Drunkenness, cured by ammonia 229
Drunkenness, prevention of .... 480
Duncan, Dr. on hydrocephalus 297
Dysphagia, case of, by Dr. Hay 300
Ear, Mr. Buchanan, on the .... 42
Earle, Mr. H. on fractures...... 58
Earle, Mr. H. plate of his fracture-bed ..................... 63
Ectropopharynx ............... 475
Edinburgh Medico-chirurgical Transactions................. 285
Effusions into the spinal canal .. 22
Elliotson, Dr. on sulphate of quinine ...................... 331
Emetine...................... 50
Emetine in violets ............ 484
Ergot of rye, where procurable.. 250
Erysipelas infantilis, quinine in.. 224
Eustachian tube, injection of.... 43
Evans, Mr. on neuralgia........ 198
Experiments, strictures on...... 182
Extirpation of the parotid gland 216
Fallopian tube, rupture of ...... 234
Fever, new theory of .......... 439
Fleurens on the brain .......... 179
Fluids of the mother, their effects on the fœtus ............... 356
Fœticide, prevalence of ........ 340
Fœticide, on the crime of ...... 340
Fœtus in the abdomen of a boy.. 180
Fothergillian medal, Mr. Bampfield ...................... 251
Fractures, Mr. H. Earle on...... 58
Fracture-bed, plate of Mr. Earle's 63
Functions of the brain.......... 179
Funis, omission of ligature to .. 350
Gairdner, Dr. on iodine ........ 104
Gaitskill, Mr. on spinal consumption...................... 456
Galvanism, for dissolving calculi 223
Ganglia, semilunar inflamed .... 187
Gangrene of the heart ........ 189
Gentianin .................... 54
Gland, parotid, extirpation of.... 216
Gland, thyroid, extirpation of .. 472
Gonorrhœa, cubebs in.......... 228
Gorget, objections to, in lithotomy 209
Green, Mr. his lectures at the college ....................... 251
Guthrie, Mr. on sloughing of the bladder ................... 235
Guthrie, Mr. on cataract ...... 396
Haden, Mr. on new remedies.... 47
Haden, Mr. death of.......... 56
Haden, Mr. on inflammatory diarrhœa .................. 112
Haden, Mr. on cut throat...... 132
Hæmorrhage, a good styptic for 142
Hawkins, Dr. on antimonial powder ....................... 227
Hay, Dr. case of dysphagia .... 300
Hayes, Mr. on blighted ovum.... 124
Hayes, Mr. on poisoning by opium 135
Head, on various injuries of .... 464
Heads of children, often swollen in parturition ............... 350
Hearing, sudden loss of ........ 180
Heart, Dr. Abercrombie on the 72
Heart, gangrene of ............ 189
Heart, on valvular diseases of the 193
Heart, malformation of ........ 301
Heermans, Dr. his case of phlegmatia dolens .............. 386
Hip-dislocation, Dr. Hunter on.. 88
Hohenloe, Prince, his miracles .. 176
Holmes, Dr. on malformation of the heart .................. 301
Hooping cough, Dr. Webster on.. 192
Hosack, Dr. on phlegmatia dolens 387
Howship, Mr. on the spine...... 8
Hunter, Dr. Adam, on dislocations 88
Hydrocephalic fever, Dr. Evanson on ....................... 364
Hydrocephalus, operated on by tapping ................... 214
Hydrocephalus, with bifid spine 297
Hydrocephalus internus ........ 479
Hydrophia, dissections of ...... 185
Hydrorachis, congenital........ 5
Hydrostatic test .............. 344
Injection of tartar emetic into the veins ..................... 484
Inflammation, Dr. Armstrong .. 118
Inflammation of the semilunar ganglia ................... 187
Infantile erysipelas, quinine in .. 224
Infanticide, various authors on .. 337
Inoculation of syphilitic virus .. 199

Insects discharged from the stomach, case of .............. 367
Intestinal tympanitis, operation for 220
Intermittents, masked.......... 485
Iodine ...................... 55
Iodine, Dr. Gairdner, on........ 104
Italian doses of medicine........ 221
Jarrold, Dr. on spinal diseases .. 303
Jaw, lower, amputation of...... 213
Kellie, Dr. on the brain ........ 285
Key, Mr. Aston on lithotomy.... 206
Kinglake, Dr. on colchicum .... 224
King and Queen's College, transactions of................. 355
Koeker, Mr. on the teeth ...... 218
Labour, premature, cases of .. 307-8
Lalor, Miss, her miraculous cure 176
Lallemand's amputation of the lower jaw ................. 213
Lallemand, Dr. on the brain.... 265
Lateral curvature of the spine .. 303
Leander, remarkable cases of scurvy in the.................... 233
Leach, Dr. versus Dr. Veitch.... 237
Lecturers, private, bye-law affecting them .................. 241
Lemon-juice, not a specific in seascurvy.................... 232
Levratt's operation for tympanites 220
Liston, Mr. operation for tetanus 217
Lithontriptor, or stone-borer.... 472
Lithotomy, Mr. Key on ........ 206
Lower jaw, amputation of...... 213
Lungs, fœtal.................. 344
Lungs, consequences of putrefaction in them ................ 345
Lungs, causes of their floating in water, distinguished........ 346-7
Lungs, weight of, a test...... 348-9
M'Clure's case of obliterated urethra ...................... 215
Madeira, its climate, &c. ...... 486
Magendie's late experiments .... 440
Malformation of the heart, case of 301
Male, Dr. on medical jurisprudence ...................... 337
Manwell, Dr. on hydrocephalus 479
Martland, Dr. on melena ...... 450
Medical remuneration.......... 509
Medulla spinalis, functions and diseases of.................. 2
Med.-Chir. Society of Edinburgh 70
Medical miracles .............. 176
Melena, Dr. Martland on ...... 450
Melena, Dr. Chisholm on ...... 231
Membrana tympani, puncture of 45
Meningeal apoplexy, Dr. Johnson on ...................... 204
Mercurial ointment, fatal result from ...................... 360
Mercury, in large dozes, in syphilis 172
Miccoli, Dr. on stibiated mercurial ointment................ 230
Miracles of Prince Hohenloe .... 176
M. Magendie, strictures on his experiments .................. 189
Mogridge, Mr. on fracture of the patella ...................... 112
Moncrief, Dr. on tuberculated peritoneum .................. 302
Morphine, salts of.............. 49
Muriate of lime, in cases of worms 226
Myelitis ...................... 33
Narcotine .................... 50
Neck, two cases of tumour removed from ................ 371
Nerves, of the teeth, denuded.... 218
Nerves, physiology of .......... 437
Nervous pregnancy .... ...... 495
Neuralgia, simulating brain disease ...................... 186
Neuralgia, Dr. Evans on ...... 198
Neuralgia, Dr. Borthwick on.... 199
Neuralgia, Dr. Uwins on........ 135
New remedies, preparation of.... 47
New Pharmacopœia............ 56
Nisbet, *versus* Parsons.......... 243
Nux vomica, resin of .......... 49
Obliteration of the urethra .... 215
Observations on a species of premature labour .............. 355
Obstruction, of the colon ...... 125
Occlusion of intestinal and urinary passages ................ 438
Olecranon, fractures of ........ 65
Olecranon, Mr. Alcock on fractures of the ................ 117
Ollivier, Dr. treatise on spinal diseases .................... 1
Omission of care to the new-born child ...................... 349
Omission of ligature to the funis 350
Operation of extirpating the parotid gland .................. 216
Opium, Mr. Battley's analysis of 253, 521
Opium, as a poison ............ 326
Opium in acute inflammation .. 118
Ovum, blighted, case of ........ 124
Ovum, blighted, Mr. Powell's case 131
Oxymurias hydrargyri (test for).. 324
Paralysis on the same side as the injury........ ............ 446
Paris, Dr. on poisons .......... 316
Paris, Dr. on infanticide........ 337
Parturition, sudden cases of .... 237
Paralysis, not a usual effect of mercury.................... 238
Parkinson, Mr. new treatment of typhus .................... 229
Paracentesis capitis............ 214
Parotid gland, extirpation of .... 216
Pathology of hooping cough.... 192
Pathology of the peritoneum.... 199
Pathology of the fluids, Dr. Stoker 144
Patella, fractures of............ 112
Peritoneum, pathological anatomy of .................... 199
Peritonitis acuta .............. 200
Peritonitis chronica............ 202
Peritoneum, tubercles of........ 302
Pharmacopœia, new............ 56
Phlegmasiæ, antimony in ...... 221

Phlegmatia dolens, essays on.... 378
Phlegmatia dolens, observations on, by Drs. Francis and Hosack 378
Phrenitis, remarkable case of, by Mr. Rhind ................ 299
Phthisis, on the tonic treatment of 256
Physiology, therapeutical ...... 176
Physiological metamorphoses.... 436
Pneumonia, large doses of antimony in .................... 221
Poisons, Dr. Paris on .......... 316
Pope, Mr. on sarsaparilla, &c. .. 47
Porrigo, ointments for the cure of 224
Powell, Mr. on twin-conception.. 130
Pregnancy with ascites ........ 492
Pregnancy, nervous ............ 495
Pregnancy, fatal constipation in.. 233
Premature labour, observations on 355
Prison-discipline .............. 245
Priapism, curious case of ...... 214
Prostate gland, section of, in lithotomy ..................... 210
Prussic acid, poisoning by ...... 506
Ptyalism, an antidote for ...... 231
Puerperal fever, Mr. Hey on .... 414
Pulvis antimonialis, experiments on 227
Purpura hæmorrhagica ........ 482
Purpura, Dr. Stoker on ........ 144
Putrefaction of the lungs........ 345
Pylorus, scirrhus of............. 462
Pylorus, scirrhus of the ........ 191
Quinine, sulphate of .......... 51
Quinina and sulphate of quinina 321
Rassori on antimony in large doses 221
Reticular texture, inflammation of 210
Rheumatism acute, Dr. Gosse on 482
Rhind, Mr. case of phrenitis .... 299
Rupture of the fallopian tube.... 234
Russel, Professor, on affection of bone ...................... 87
Ryan, Dr. on angina pectoris.... 203
Sanders, Dr. on phthisis........ 256
Sarsaparilla, Mr. Pope on ...... 47
Scirrhus of the pylorus ........ 191
Scoutteten on the peritoneum .. 199
Scudamore, Dr. on the blood.... 137
Searle, Mr. on compression...... 516
Sea-scurvy not always cured by lemon-juice ................. 231
Shaw, Mr. on spinal diseases .... 303
Shaw, Mr. on stricture of the urethra ..................... 475
Shearman, Dr. on debility ...... 391
Simulated diseases ............ 508
Sizy blood, on the nature of .... 139
Sloughing of the bladder, post partum ................... 235
Smith, Dr. on infanticide ...... 337
Speech, sudden loss of.......... 180
Spinal marrow, on diseases of.... 1
Spinal marrow, on functions of .. 2
Spinal marrow, on compression of 13
Spinal arachnitis .............. 32
Spinal diseases ................ 303
Spinal consumption............. 456
Spine, Dr. Dods, on the ........ 93
Spleen, Dr. Vetch on .......... 460
Sprague, Mr. formula for porrigo 224
Stevenson, Mr. on cataract...... 396
Stewart, Dr. on phthisis ........ 256
Stethoscope, in diagnosis of aneurisms ..................... 183
Stoker, Dr. on the pathology of the fluids .................. 144
Stibiated mercurial ointment.... 230
Strichnine .................... 49
Stricture of the urethra, Mr. Shaw 475
Stricture of the urethra,—Mr. Arnott ..................... 474
Styptic, Dr. Scudamore's........ 142
Suffocation of children ........ 351
Sulphate of copper, as a test for arsenic ................... 322
Sulphate of quinine in erysipelas 224
Sulphate of quinine in hemicrania 226
Sulphate of quinine, remarks on 487
Superfœtation, remarkable case of 181
Surgeons, college of, their late bye-law ...................... 241
Syncope, its effects on inflammation ...................... 141
Syphilitic virus inoculated ...... 199
Syphilis, Mr. Boyle's treatise on.. 172
Tartrite of antimony in large doses 483
Teeth, nerves of, denuded ...... 218
Tetanus traumaticus, amputation 217
Tetanus, on the use of tobacco in 90
Therapeutical physiology........ 176
Tinea, Dr. Crampton on ........ 359
Tinea, treated by warm bath, &c. 360
Tobacco in tetanus ............ 90
Tommasini, on English medicine 160
Tongue, remarks on the........ 250
Transactions of the Ed. Med.-Chir. Society ................... 70
Transactions of King and Queen's College &c. ................ 355
Tread-Mill, remarks on ........ 245
Tubercular disease of the peritoneum ..................... 302
Tumour, remarkable, of a nerve 18
Tumour of the neck, cases of .. 371
Turn of life .................. 496
Twins, curious case of, Mr. Powell 131
Tympanites intestinalis ........ 220
Typhus fever, new treatment of 229
Urethra, obliteration of ........ 215
Uwins, Dr. on aphonia ........ 135
Valvular diseases of the heart.... 193
Variola after vaccination........ 366
Veitch, Dr. ill-treated .......... 237
Veratrine .................... 53
Vetch, Dr. on the spleen........ 460
Warm bath in tinea............ 360
Weight of new-born children .... 343
Weight of the lungs a test of infanticide.................. 348-9
Wounds of the spinal marrow .. 8
Zinn, his case of phlegmatia dolens 379

